# Correction: Late-season surveys to document seed rain potential of Palmer amaranth (*Amaranthus palmeri*) and waterhemp (*Amaranthus tuberculatus*) in Texas cotton

**DOI:** 10.1371/journal.pone.0249463

**Published:** 2021-03-30

**Authors:** The PLOS ONE Staff

In Fig 2 the Y-axis and the legend cannot be seen. The authors have provided a corrected version here. The publisher apologizes for the error.

**Fig 2:**
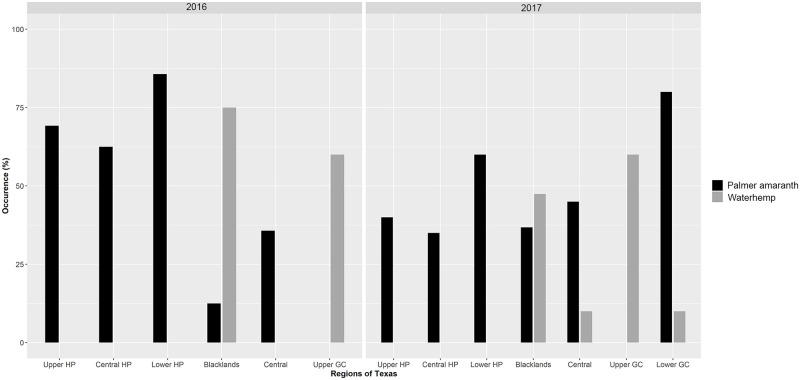
Percent of surveyed fields infested with *A*. *palmeri* and *A*. *tuberculatus* escapes in the major cotton-producing regions in Texas in 2016 and 2017. The Lower GC region was surveyed only in 2017. Abbreviations: HP, High Plains; GC, Gulf Coast.
