# Supplementary material for: Late-season surveys to document seed rain potential of Palmer amaranth (Amaranthus palmeri) and waterhemp (Amaranthus tuberculatus) in Texas cotton
Source: PLoS One. 2020 Jun 8;15(6):e0226054. doi: 10.1371/journal.pone.0226054 (PMC7279589; doi:10.1371/journal.pone.0226054)
Supplement: S1 Table — (DOCX) [file pone.0226054.s001.docx]

**S1_Table. Latitude and longitude of the cotton fields (primary survey sites) surveyed in 2016 and 2017 in Texas.**

| **Year** | **Region**^a^ | **Field** | **Latitude** | **Longitude** |
| --- | --- | --- | --- | --- |
| 2016 | Blacklands | 1 | 30.707131 | -96.552426 |
| 2016 | Blacklands | 2 | 30.716116 | -96.556209 |
| 2016 | Blacklands | 3 | 30.37041 | -96.21226 |
| 2016 | Blacklands | 4 | 30.48621 | -96.36005 |
| 2016 | Blacklands | 5 | 30.50589 | -96.38915 |
| 2016 | Blacklands | 6 | 30.520685 | -96.39891 |
| 2016 | Blacklands | 7 | 31.15754 | -96.55079 |
| 2016 | Blacklands | 8 | 31.17044 | -96.54915 |
| 2016 | Blacklands | 9 | 31.18022 | -96.56909 |
| 2016 | Blacklands | 10 | 31.25455 | -97.03137 |
| 2016 | Blacklands | 11 | 31.25955 | -97.02248 |
| 2016 | Blacklands | 12 | 31.2765 | -97.0294 |
| 2016 | Blacklands | 13 | 30.42953 | -97.18418 |
| 2016 | Blacklands | 14 | 30.40089 | -97.30282 |
| 2016 | Blacklands | 15 | 30.33006 | -97.30281 |
| 2016 | Central HP | 1 | 34.20473 | -101.44462 |
| 2016 | Central HP | 2 | 34.19707 | -101.44264 |
| 2016 | Central HP | 3 | 34.19704 | -101.44261 |
| 2016 | Central HP | 4 | 34.17039 | -101.42555 |
| 2016 | Central HP | 5 | 34.16666 | -101.4254 |
| 2016 | Central HP | 6 | 34.16418 | -101.43583 |
| 2016 | Central HP | 7 | 34.16205 | -101.43543 |
| 2016 | Central HP | 8 | 34.13382 | -101.4667 |
| 2016 | Central HP | 9 | 34.09978 | -101.47 |
| 2016 | Central HP | 10 | 34.052 | -101.50145 |
| 2016 | Central HP | 11 | 33.59549 | -101.5174 |
| 2016 | Central HP | 12 | 34.14009 | -101.4377 |
| 2016 | Central HP | 13 | 34.10537 | -101.45852 |
| 2016 | Central HP | 14 | 34.10536 | -101.45352 |
| 2016 | Central Texas | 1 | 29.14879 | -99.37355 |
| 2016 | Central Texas | 2 | 29.151 | -99.36538 |
| 2016 | Central Texas | 3 | 29.1794 | -99.30644 |
| 2016 | Central Texas | 4 | 29.18264 | -99.27811 |
| 2016 | Central Texas | 5 | 29.173 | -99.27685 |
| 2016 | Central Texas | 6 | 29.17673 | -99.27605 |
| 2016 | Central Texas | 7 | 29.17239 | -99.27687 |
| 2016 | Central Texas | 8 | 29.19907 | -99.21644 |
| 2016 | Central Texas | 9 | 29.20211 | -99.1488 |
| 2016 | Central Texas | 10 | 29.20224 | -99.12605 |
| 2016 | Central Texas | 11 | 29.15775 | -98.52511 |
| 2016 | Central Texas | 12 | 29.15754 | -98.51324 |
| 2016 | Central Texas | 13 | 29.19492 | -98.47213 |
| 2016 | Central Texas | 14 | 29.28853 | -95.50077 |
| 2016 | Lower HP | 1 | 33.28858 | -102.0365 |
| 2016 | Lower HP | 2 | 33.28185 | -102.04692 |
| 2016 | Lower HP | 3 | 33.2857 | -102.04782 |
| 2016 | Lower HP | 4 | 33.27641 | -102.0554 |
| 2016 | Lower HP | 5 | 33.24692 | -102.09907 |
| 2016 | Lower HP | 6 | 33.20449 | -102.12279 |
| 2016 | Lower HP | 7 | 33.12869 | -102.1646 |
| 2016 | Lower HP | 8 | 33.14234 | -102.16469 |
| 2016 | Lower HP | 9 | 33.10968 | -102.3366 |
| 2016 | Lower HP | 10 | 33.08433 | -102.44973 |
| 2016 | Lower HP | 11 | 33.07383 | -102.15818 |
| 2016 | Lower HP | 12 | 33.10857 | -102.14266 |
| 2016 | Lower HP | 13 | 33.26903 | -101.40122 |
| 2016 | Lower HP | 14 | 33.2688 | -101.4207 |
| 2016 | Upper GC | 1 | 29.34658 | -95.48096 |
| 2016 | Upper GC | 2 | 29.33471 | -95.50029 |
| 2016 | Upper GC | 3 | 29.33428 | -95.50729 |
| 2016 | Upper GC | 4 | 29.3341 | -95.51332 |
| 2016 | Upper GC | 5 | 29.34404 | -95.52518 |
| 2016 | Upper GC | 6 | 29.37855 | -95.5761 |
| 2016 | Upper GC | 7 | 29.37073 | -96.01996 |
| 2016 | Upper GC | 8 | 29.27354 | -96.04329 |
| 2016 | Upper GC | 9 | 29.18929 | -95.59597 |
| 2016 | Upper GC | 10 | 29.20076 | -95.46243 |
| 2016 | Upper GC | 11 | 29.049 | -95.44688 |
| 2016 | Upper GC | 12 | 29.24472 | -95.45215 |
| 2016 | Upper GC | 13 | 29.25645 | -95.45357 |
| 2016 | Upper GC | 14 | 29.48542 | -95.712884 |
| 2016 | Upper GC | 15 | 29.31809 | -95.82812 |
| 2016 | Upper HP | 1 | 36.0663 | -102.23065 |
| 2016 | Upper HP | 2 | 36.17951 | -102.0349 |
| 2016 | Upper HP | 3 | 36.17951 | -102.04562 |
| 2016 | Upper HP | 4 | 36.17319 | -102.03749 |
| 2016 | Upper HP | 5 | 36.16599 | -102.03739 |
| 2016 | Upper HP | 6 | 36.15307 | -102.03734 |
| 2016 | Upper HP | 7 | 36.08552 | -101.57254 |
| 2016 | Upper HP | 8 | 36.07075 | -101.57276 |
| 2016 | Upper HP | 9 | 34.2751 | -101.46664 |
| 2017 | Blacklands | 1 | 30.611021 | -96.318495 |
| 2017 | Blacklands | 2 | 30.704955 | -96.551759 |
| 2017 | Blacklands | 3 | 30.789745 | -96.599119 |
| 2017 | Blacklands | 4 | 30.79784 | -96.597018 |
| 2017 | Blacklands | 5 | 30.815026 | -96.594414 |
| 2017 | Blacklands | 6 | 31.147947 | -96.823767 |
| 2017 | Blacklands | 7 | 30.601573 | -97.301396 |
| 2017 | Blacklands | 8 | 30.604454 | -97.286817 |
| 2017 | Blacklands | 9 | 30.572919 | -97.334788 |
| 2017 | Blacklands | 10 | 30.570375 | -97.33863 |
| 2017 | Blacklands | 11 | 30.500311 | -97.400701 |
| 2017 | Blacklands | 12 | 30.499345 | -97.388307 |
| 2017 | Blacklands | 13 | 30.520937 | -97.362209 |
| 2017 | Blacklands | 14 | 30.608158 | -97.388078 |
| 2017 | Blacklands | 15 | 30.607811 | -97.398723 |
| 2017 | Blacklands | 16 | 30.653394 | -97.383267 |
| 2017 | Blacklands | 17 | 30.745563 | -97.358165 |
| 2017 | Blacklands | 18 | 30.752247 | -97.380008 |
| 2017 | Central HP | 1 | 34.264269 | -101.70903 |
| 2017 | Central HP | 2 | 34.270488 | -101.70855 |
| 2017 | Central HP | 3 | 34.277821 | -101.70848 |
| 2017 | Central HP | 4 | 34.27837 | -101.68993 |
| 2017 | Central HP | 5 | 34.448113 | -101.76471 |
| 2017 | Central HP | 6 | 34.470615 | -101.7684 |
| 2017 | Central HP | 7 | 34.53852 | -102.0881 |
| 2017 | Central HP | 8 | 34.538928 | -102.13757 |
| 2017 | Central HP | 9 | 34.539829 | -102.19648 |
| 2017 | Central HP | 10 | 34.307967 | -102.30442 |
| 2017 | Central HP | 11 | 34.011155 | -102.33127 |
| 2017 | Central HP | 12 | 34.010983 | -102.36555 |
| 2017 | Central HP | 13 | 33.810934 | -102.08585 |
| 2017 | Central HP | 14 | 33.824592 | -102.05091 |
| 2017 | Central HP | 15 | 33.824117 | -101.85396 |
| 2017 | Central HP | 16 | 33.794977 | -101.85414 |
| 2017 | Central HP | 17 | 33.727069 | -101.84233 |
| 2017 | Central HP | 18 | 33.687162 | -101.75009 |
| 2017 | Central HP | 19 | 33.694436 | -101.73743 |
| 2017 | Central HP | 20 | 33.665582 | -101.3325 |
| 2017 | Central Texas | 1 | 29.24643 | -99.64783 |
| 2017 | Central Texas | 2 | 29.26188 | -99.64475 |
| 2017 | Central Texas | 3 | 29.31496 | -99.59584 |
| 2017 | Central Texas | 4 | 29.29464 | -99.45606 |
| 2017 | Central Texas | 5 | 29.29455 | -99.44989 |
| 2017 | Central Texas | 6 | 29.456 | -99.44658 |
| 2017 | Central Texas | 7 | 29.29449 | -99.44168 |
| 2017 | Central Texas | 8 | 29.31437 | -99.37486 |
| 2017 | Central Texas | 9 | 29.33761 | -99.36077 |
| 2017 | Central Texas | 10 | 29.31633 | -99.3477 |
| 2017 | Central Texas | 11 | 29.33657 | -99.20656 |
| 2017 | Central Texas | 12 | 29.27385 | -99.27461 |
| 2017 | Central Texas | 13 | 29.32894 | -99.19682 |
| 2017 | Central Texas | 14 | 29.3146 | -99.19953 |
| 2017 | Central Texas | 15 | 29.30779 | -99.13711 |
| 2017 | Central Texas | 16 | 29.30776 | -99.14028 |
| 2017 | Central Texas | 17 | 29.30727 | -99.16798 |
| 2017 | Central Texas | 18 | 29.34943 | -98.85603 |
| 2017 | Central Texas | 19 | 29.34586 | -98.86408 |
| 2017 | Lower GC | 1 | 28.24132 | -97.79438 |
| 2017 | Lower GC | 2 | 28.24316 | -97.78774 |
| 2017 | Lower GC | 3 | 28.25749 | -97.82752 |
| 2017 | Lower GC | 4 | 28.19074 | -97.81403 |
| 2017 | Lower GC | 5 | 28.17838 | -97.80349 |
| 2017 | Lower GC | 6 | 28.15382 | -97.76936 |
| 2017 | Lower GC | 7 | 27.89752 | -97.86817 |
| 2017 | Lower GC | 8 | 27.45241 | -97.86139 |
| 2017 | Lower GC | 9 | 27.57962 | -97.74081 |
| 2017 | Lower GC | 10 | 27.57575 | -97.63718 |
| 2017 | Lower GC | 11 | 27.61065 | -97.84671 |
| 2017 | Lower GC | 12 | 27.66085 | -97.75401 |
| 2017 | Lower GC | 13 | 27.63744 | -97.45721 |
| 2017 | Lower GC | 14 | 27.66332 | -97.50328 |
| 2017 | Lower GC | 15 | 27.94999 | -97.65648 |
| 2017 | Lower GC | 16 | 27.97901 | -97.59752 |
| 2017 | Lower GC | 17 | 27.95482 | -97.57136 |
| 2017 | Lower GC | 18 | 27.94239 | -97.55657 |
| 2017 | Lower GC | 19 | 27.95078 | -97.52275 |
| 2017 | Lower GC | 20 | 27.97956 | -97.52217 |
| 2017 | Lower HP | 1 | 30.795213 | -97.427751 |
| 2017 | Lower HP | 2 | 32.863383 | -102.79493 |
| 2017 | Lower HP | 3 | 32.908175 | -102.79476 |
| 2017 | Lower HP | 4 | 32.915027 | -102.75641 |
| 2017 | Lower HP | 5 | 32.915113 | -102.66525 |
| 2017 | Lower HP | 6 | 32.925429 | -102.46811 |
| 2017 | Lower HP | 7 | 32.920757 | -102.4681 |
| 2017 | Lower HP | 8 | 32.875094 | -102.46816 |
| 2017 | Lower HP | 9 | 32.907957 | -102.41631 |
| 2017 | Lower HP | 10 | 32.912022 | -102.26121 |
| 2017 | Lower HP | 11 | 32.912229 | -102.12277 |
| 2017 | Lower HP | 12 | 32.872627 | -102.09813 |
| 2017 | Lower HP | 13 | 32.751297 | -101.96732 |
| 2017 | Lower HP | 14 | 32.852763 | -101.89224 |
| 2017 | Lower HP | 15 | 33.151329 | -101.7891 |
| 2017 | Lower HP | 16 | 33.469261 | -102.07803 |
| 2017 | Lower HP | 17 | 33.460515 | -102.09214 |
| 2017 | Lower HP | 18 | 33.326285 | -102.21229 |
| 2017 | Lower HP | 19 | 33.311807 | -102.23091 |
| 2017 | Lower HP | 20 | 33.051429 | -102.68111 |
| 2017 | Upper GC | 1 | 28.814313 | -96.696195 |
| 2017 | Upper GC | 2 | 28.859198 | -96.645574 |
| 2017 | Upper GC | 3 | 28.884418 | -96.659965 |
| 2017 | Upper GC | 4 | 28.996874 | -96.63007 |
| 2017 | Upper GC | 5 | 28.996654 | -96.630541 |
| 2017 | Upper GC | 6 | 29.01049 | -96.574009 |
| 2017 | Upper GC | 7 | 29.064264 | -96.51462 |
| 2017 | Upper GC | 8 | 29.076029 | -96.510769 |
| 2017 | Upper GC | 9 | 29.070131 | -96.414258 |
| 2017 | Upper GC | 10 | 29.247961 | -96.306576 |
| 2017 | Upper GC | 11 | 29.249346 | -93.304008 |
| 2017 | Upper GC | 12 | 29.270672 | -96.316321 |
| 2017 | Upper GC | 13 | 29.303899 | -96.199699 |
| 2017 | Upper GC | 14 | 29.347184 | -96.090506 |
| 2017 | Upper GC | 15 | 29.355262 | -96.045988 |
| 2017 | Upper GC | 16 | 29.399073 | -96.1479 |
| 2017 | Upper GC | 17 | 29.504344 | -96.072443 |
| 2017 | Upper GC | 18 | 29.569626 | -96.076214 |
| 2017 | Upper GC | 19 | 29.573015 | -96.083404 |
| 2017 | Upper GC | 20 | 29.577169 | -96.069863 |
| 2017 | Upper HP | 1 | 35.868939 | -102.38108 |
| 2017 | Upper HP | 2 | 35.867581 | -102.34179 |
| 2017 | Upper HP | 3 | 35.866473 | -102.32147 |
| 2017 | Upper HP | 4 | 35.866745 | -102.23485 |
| 2017 | Upper HP | 5 | 35.869948 | -102.26411 |
| 2017 | Upper HP | 6 | 36.139651 | -102.6481 |
| 2017 | Upper HP | 7 | 36.139779 | -102.66582 |
| 2017 | Upper HP | 8 | 36.136435 | -102.6255 |
| 2017 | Upper HP | 9 | 36.171767 | -102.6888 |
| 2017 | Upper HP | 10 | 36.1689 | -102.87532 |
| 2017 | Upper HP | 11 | 36.082358 | -102.76524 |
| 2017 | Upper HP | 12 | 36.082039 | -102.74924 |
| 2017 | Upper HP | 13 | 36.079269 | -102.74924 |
| 2017 | Upper HP | 14 | 36.284436 | -102.15198 |
| 2017 | Upper HP | 15 | 36.259137 | -102.15184 |

^a^Abbreviation: HP, High Plains; and GC, Gulf Coast
